# Circ_0000140 restrains the proliferation, metastasis and glycolysis metabolism of oral squamous cell carcinoma through upregulating CDC73 via sponging miR-182-5p

**DOI:** 10.1186/s12935-020-01501-7

**Published:** 2020-08-26

**Authors:** Jia Guo, Yuanyuan Su, Meng Zhang

**Affiliations:** Stomatological Center, The First Affiliated Hospital of Zhengzhou University, Zhengzhou, 450052 Henan China

**Keywords:** OSCC, circ_0000140, miR-182-5p, CDC73

## Abstract

**Background:**

Oral squamous cell carcinoma (OSCC) is a more common cancer in the world. Emerging evidence suggests that circular RNAs (circRNAs) participate in the progression of OSCC. However, the role of circ_0000140 in OSCC is still unknown.

**Methods:**

The expression of circ_0000140 and microRNA-182-5p (miR-182-5p) were assessed by quantitative real-time polymerase chain reaction (qRT-PCR). Also, cell proliferation, migration and invasion were measured by colony formation and transwell assays, respectively. Western blot (WB) analysis was used to test the levels of proliferation, metastasis and glycolysis metabolism-related proteins as well as cell division cycle 73 (CDC73) protein. Further, the extracellular acidification rate (ECAR) of cells was detected by the Seahorse XF Extracellular Flux Analyzer. The lactate acid level of cells was tested by Lactate Assay Kit. Moreover, dual-luciferase reporter was used to verify the interaction between miR-182-3p and circ_0000140 or CDC73, and RNA immunoprecipitation (RIP) assay was employed to further confirm the relationship between miR-182-3p and circ_0000140. In addition, mice xenograft models were built to measure the effect of circ_0000140 on OSCC tumor growth in vivo.

**Results:**

Circ_0000140 was lowly expressed in OSCC, and its overexpression hindered proliferation, migration, invasion and glycolysis metabolism in OSCC cells. MiR-182-5p could be sponged by circ_0000140, and its mimic could invert the suppression of circ_0000140 overexpression on OSCC progression. CDC73 could be targeted by miR-182-3p, and its silencing could reverse the inhibition of miR-182-3p inhibitor on OSCC progression. Further, overexpressed circ_0000140 reduced the OSCC tumor growth in vivo.

**Conclusions:**

Circ_0000140 might play an anti-cancer role in OSCC, which provided a novel target for clinical therapy of OSCC.

## Background

Oral squamous cell carcinoma (OSCC) is a common type of oral cancer and belongs to the category of malignant tumors [1, 2]. At present, the incidence of OSCC is still on the rise, and the 5-year survival rate is still about 50% [3]. In addition to local hyperplasia and aggression of surrounding tissues, OSCC often causes corresponding lymphatic metastasis in the neck [4, 5]. OSCC patients often present with pain and dysphagia, which can seriously affect their quality of life [6]. Therefore, it is urgent to clarify the mechanism affecting the development of OSCC.

Circular RNAs (circRNAs) are a class of RNAs that have been studied a lot in recent years and have received extensive attention due to their unique covalently closed structure [7]. Many studies have demonstrated that circRNAs can be involved in the proliferation and metastasis of cancer cells to play a pro-cancer or anti-cancer effect in cancer progressions, such as circFMN2, circFBXL5 and circGRAMD18 [8–10]. In OSCC, hsa_circ_0004491 was found to be lowly expressed in OSCC, and its knockdown could enhance the metastasis ability of OSCC [11]. Hsa_circ_100533 had been confirmed to be downregulated in OSCC and had a significant inhibitory effect on the proliferation and migration of OSCC [12]. Circ_0000140 is a newly discovered circRNA with significantly low expression in OSCC patients [13]. A recent study had shown that low circ_0000140 expression was significantly associated with the poor prognosis in OSCC patients and could function as a potential biomarker for OSCC [14]. Therefore, circ_0000140 might be an important target for the treatment of OSCC, and its role and mechanism in the progression of OSCC deserved further investigation.

MicroRNAs (miRNAs) have been shown to be widely involved in the regulation of many diseases, including cancer [15, 16]. The reason that miRNAs have been widely studied is that circRNA can function as “miRNA sponges” to regulate downstream gene expression, thus participating in the regulation of cancer progression [17, 18]. MiR-182-5p is a widely highly expressed miRNA in many cancers and can promote proliferation and metastasis in cancer cells, such as breast cancer and hepatocellular carcinoma [19, 20]. Studies have shown that miR-182-5p is upregulated in OSCC and is related to OSCC metastasis [21, 22]. Cell division cycle 73 (CDC73) has been shown to be an important tumor suppressor and is down-regulated in OSCC [23, 24]. Therefore, studies on miR-182-5p and CDC73 will help us to understand the molecular mechanism of OSCC progress.

Here, we mainly discuss the role of circ_0000140 in OSCC, and clarify its mechanism through bioinformatics prediction and experimental verification. The proposed circ_0000140 function added new evidence for its role as a biomarker of OSCC.

## Materials and methods

### Tissue samples

OSCC tissues (OSCC) and non-tumor margin tissues (Normal) of 60 OSCC patients were obtained from The First Affiliated Hospital of Zhengzhou University. These included 27 patients with lymph node metastasis (Yes) and 33 patients without lymph node metastasis (No). All tissues were removed before treatment and stored at – 80 °C. Each patient provided written informed consent. Our study protocols were approved by the Ethics Committee of The First Affiliated Hospital of Zhengzhou University.

### Cell culture

OSCC cell lines (CAL-27, SCC-4, SCC-9 and SCC-25) were bought from the American Type Culture Collection (ATCC, Manassas, VA, USA). Human normal oral epithelial keratinocytes (HOK) were obtained from Tongpai (Shanghai, China). All cells were cultured in Dulbecco’s modified Eagle’s medium (DMEM; Gibco, Carlsbad, CA, USA) and supplemented with 10% fetal bovine serum (FBS; Gibco), 1% penicillin-streptomycin (100 U/mL–100 µg/mL; Solarbio, Beijing, China) at 37 °C with 5% CO_2_ incubator.

### Quantitative real-time polymerase chain reaction (qRT-PCR)

TRIzol (Invitrogen, Carlsbad, CA, USA) was used to extract total RNAs. Nanodrop (Thermo Fisher Scientific, Waltham, MA, USA) was used to evaluate the purity of RNA. The 260/280 absorption ratio of RNA ranged from 2 to 2.1, indicating good purity of RNA. Then, the gel electrophoresis was performed to detect the integrity of RNA, which could produce clear 28S and 18S rRNA bands, indicating good integrity of RNA. Next, 2 µg of extracted total RNA was reversely transcribed into cDNA using Transcriptor Universal cDNA Master (Roche, Basel, Switzerland). qRT-PCR was performed using SYBR Green (Solarbio). The amplification process was as follows: denaturation at 95 °C for 5 min, followed by 40 cycles of at 95 °C for 15 s, annealing at 55 °C for 30 s, and extension at 60 °C for 1 min. The results were normalized using 18S ribosomal RNA (rRNA) or U6 and expressed using the 2^−ΔΔCt^ methods. All primers were bought from RiboBio (Guangzhou, China) and the sequences were presented as below: circ_0000140, F 5′-TCCCTACGGAGTACCAAGCA-3′, R 5′-GCAAATAGGAGCAGCCTGGA-3′; KIAA0907, F 5′-AGACTCAAGACGAGGTGAGGAG-3′, R 5′-ATGACCCAAACCCACTAATAAA-3′; 18 s rRNA, F 5′-AGGGCTAATACATGTTCGAGGCCATTT-3′, R 5′-TCCCTCTAAGAAGCGATAACGGGACAGT-3′; miR-182-5p, F 5′-ATCACTTTTGGCAATGGTAGAACT-3′, R 5′-TATGGTTTTGACGACTGTGTGAT-3′; U6, F 5′-CTCGCTTCGGCAGCACA-3′; R 5′-AACGCTTCACGAATTTGCGT-3′.

### CircRNA identification and localization

After RNAs were extracted from CAL-27 and SCC-4 cells, the extracted RNAs were treated with Ribonuclease R (RNase R; Duma, Shanghai, China) and then performed qRT-PCR to detect the expression levels of circ_0000140 and KIAA0907 to evaluate the circular characteristic of circ_0000140.

Cytoplasmic & Nuclear RNA Purification Kit (Norgen Biotek, Thorold, ON, Canada) was used to isolate and extract the cytoplasm and nuclear RNA of CAL-27 and SCC-4 cells. Then, qRT-PCR was used to measure the circ_0000140, 18 s rRNA and U6 expression in nuclear and cytoplasm. 18 s rRNA and U6 were served as the cytoplasm internal control and nuclear internal control, respectively.

### Cell transfection

Circ_0000140 overexpression vector (circ_0000140, F, 5′-GCCGGCATTACCTACTGGAG-3′, R, 5′-TGCAAATAGGAGCAGCCTGG-3′), miR-182-5p mimic (miR-182-5p, 5′-UUUGGCAAUGGUAGAACUCACACCG-3′) and inhibitor (anti-miR-182-5p, 5′-TGTGAGTTCTACCATTGCCAA-3′), small interfering RNA (siRNA) against CDC73 (si-CDC73, 5′-GAGUACUACACAUUGGAUUCC-3′) and or their negative controls (vector, miR-NC, anti-NC and si-NC) were synthesized by RiboBio. Cell transfection was performed using Lipofectamine 3000 (Invitrogen).

### Colony formation assay

CAL-27 and SCC-4 cells were seeded into 6-well plates. After transfection, cells were grown for 10 days. After that, CAL-27 and SCC-4 cells were fixed with methanol and stained with crystal violet. Randomly observed 6 fields and counted the number of colonies (> 50 cells).

### Transwell assay

Transwell chambers (Corning Inc., Corning, NY, USA) were used for this experiment. The upper chambers were coated with Matrigel (BD Biosciences, San Jose, CA, USA) to measure invasion, while non-coated to detect migration. Briefly, after transfection for 24 h, CAL-27 and SCC-4 cells were harvested and seeded into the upper chambers containing serum-free medium, while the lower chambers contained serum medium. After incubation for 24 h, cells were fixed with methanol and stained with crystal violet. Randomly observed 6 fields and counted the number of migrated and invaded cells.

### Western blot (WB) analysis

Total proteins were lysed using RIPA buffer (Beyotime, Shanghai, China) and quantified using BCA Kit (Beyotime). Protein samples were separated in sodium dodecyl sulfate-polyacrylamide gel electrophoresis (SDS-PAGE) gels and transferred to polyvinylidene fluoride (PVDF) membranes (Millipore, Billerica, MA, USA). Skim milk (5%) was used to block the membranes. After that, the membranes were incubated with primary antibodies against ki67 (1:200, Bioss, Beijing, China), matrix metalloproteinase-2 (MMP-2, 1:1,000, Bioss), MMP-9 (1:500, Bioss), glucose transporter 1 (GLUT1; 1:1,000, Abcam, Cambridge, MA, USA), lactate dehydrogenase A (LDHA; 1:5,000, Abcam), CDC73 (1:1,000, Invitrogen) or glyceraldehyde 3-phosphate dehydrogenase (GAPDH; 1:1,000, Abcam) at 4 °C overnight, and then incubated with secondary antibody (1:2,000, Abcam) for 1 h. Protein signals were visualized using enhanced chemiluminescence (Yeasen, Shanghai, China). The protein levels were analyzed using ImageJ software.

### Extracellular acidification rate (ECAR) detection

This experiment was performed using the Seahorse XF Extracellular Flux Analyzer (Seahorse Bioscience, Billerica, MA, USA). Briefly, CAL-27 and SCC-4 cells were successively treated with glucose, oligomycin (OM) and 2-deoxy glucose (2-DG) at the indicated points after transfection for 48 h, and the ECAR of cells was monitored.

### Measurement of lactate acid level

After transfection for 48 h, the lactate acid level of CAL-27 and SCC-4 cells were measured by Lactate Assay Kit (BioVision, Milpitas, CA, USA) according to the manufacturer’s instructions.

### Dual-luciferase reporter assay

The sequences of wild-type (WT) and mutant-type (MUT) circ_0000140 were cloned into the pmirGLO reporter vectors (Enzyme Research, Shanghai, China) to construct circ_0000140 WT and circ_0000140 MUT reporter vectors. Also, CDC73 WT and CDC73 MUT reporter vectors were built in the same way. CAL-27 and SCC-4 cells were co-transfected with the reporter vectors and miR-182-5p mimic or miR-NC using Lipofectamine 3000. The luciferase activity of cells was measured by the Dual-Luciferase Reporter Assay Kit (Beyotime).

### RNA immunoprecipitation (RIP) assay

RIP Kit (Millipore) was used to perform this experiment. CAL-27 and SCC-4 cells were lysed by RIP buffer and cell lysates were collected. Then, magnetic beads conjugated with argonaute2 antibody (anti-Ago2) or immunoglobulin G antibody (anti-IgG) were added into cell lysates and incubated overnight at 4 °C. After the RNA was purified, the enrichment of circ_0000140 and miR-182-5p was tested by qRT-PCR.

### Mice xenograft models

CAL-27 cells (2 × 10^6^) stably transfected with circ_0000140 overexpression vector or vector were subcutaneously injected into BALB/c nude mice (n = 06/group; Cavens, Changzhou, China). Tumor length and width were measured once every 7 days and calculated the tumor volume using the formula: volume (mm^3^) = width^2^ × length/2. After 28 days, the mice were sacrificed and removed the tumors to perform further experiments. Animal studies were performed in compliance with the ARRIVE guidelines and the Basel Declaration. Experimental procedures were approved by the Institutional and Local Committee on the Care and Use of Animals of The First Affiliated Hospital of Zhengzhou University. All animals received humane care according to the National Institutes of Health (USA) guidelines.

### Statistical analysis

Statistical analyses were determined by GraphPad Prism 5.0 software (GraphPad Software, San Diego, CA, USA). All data were displayed as the mean ± standard deviation. The statistical differences were calculated by student’s *t*-test or one-way analysis of variance. The *P*-value < 0.05 was considered as statistically significant.

## Results

### Circ_0000140 was lowly expressed in OSCC and was associated with lymph node metastasis of OSCC

**Fig. 1:**
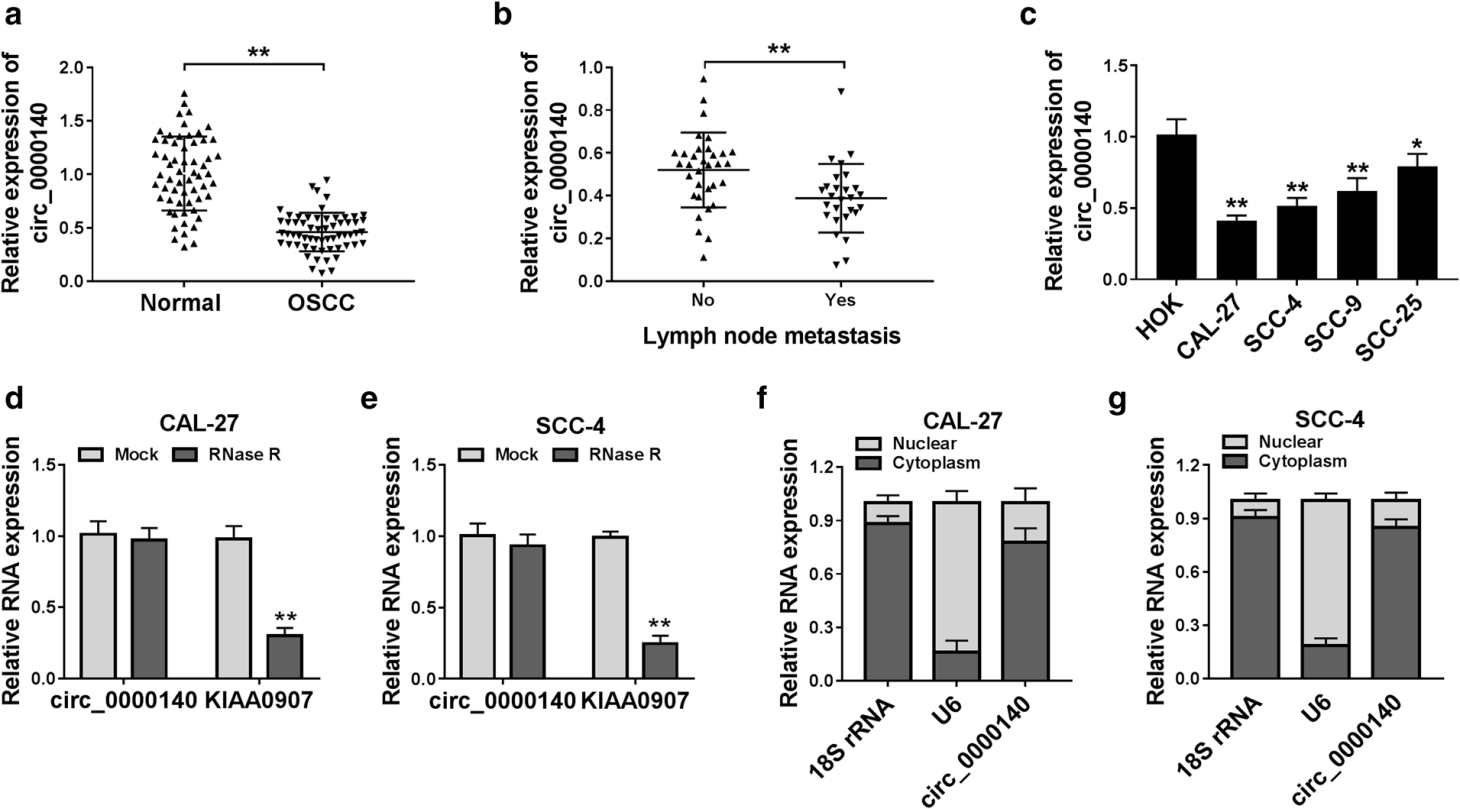
The expression of circ_0000140 in OSCC tissues and cells. **a** The expression of circ_0000140 in OSCC tissues (OSCC) and adjacent normal mucosal tissues (Normal) was detected by qRT-PCR. **b** The circ_0000140 expression in OSCC tissues with or without lymph node metastasis (Yes or No) was measured by qRT-PCR. **c** qRT-PCR was used to test the circ_0000140 expression in OSCC cell lines (CAL-27, SCC-4, SCC-9 and SCC-25) and HOK cells. **d**, **e** The relative expression levels of circ_0000140 and KIAA0907 in CAL-27 and SCC-4 cells were assessed by qRT-PCR after treatment with RNase R. **f**, **g** The relative expression levels of circ_0000140, U6 and 18 s rRNA in the nuclear and cytoplasmic of CAL-27 and SCC-4 cells were detected by qRT-PCR. * *P* < 0.05, ** *P* < 0.01

Firstly, we tested the expression of circ_0000140 in OSCC tissues. As presented in Fig. 1a, compared to non-tumor margin tissues, the circ_0000140 expression was remarkably down-regulated in OSCC tissues. Besides, we also found that in OSCC tissues with lymph node metastasis, circ_0000140 expression was significantly lower than that in OSCC tissues without lymph node metastasis (Fig. 1b). Moreover, we measured the expression of circ_0000140 in OSCC cell lines (CAL-27, SCC-4, SCC-9 and SCC-25) using qRT-PCR and discovered that circ_0000140 also was under-expressed in OSCC cell lines (especially CAL-27 and SCC-4) compared with that in HOK cells (Fig. 1c). To confirm the circular characteristic of circ_0000140, we used RNase R to perform qRT-PCR. The results showed that circ_0000140 could resist the action of RNase R, while linear RNA KIAA0907 could be digested by RNase R, indicating that circ_0000140 was circular (Fig. 1d, e). Furthermore, we assessed the subcellular distribution of circ_0000140 in CAL-27 and SCC-4 cells and found that circ_0000140 was mainly distributed in the cytoplasm of OSCC cells, suggesting that circ_0000140 might be mainly involved in post-transcriptional regulation (Fig. 1f, g). Therefore, we concluded that circ_0000140 was a circular transcript and might play an important role in OSCC progression.

### Circ_0000140 overexpression suppressed the proliferation, migration and invasion of OSCC cells

**Fig. 2:**
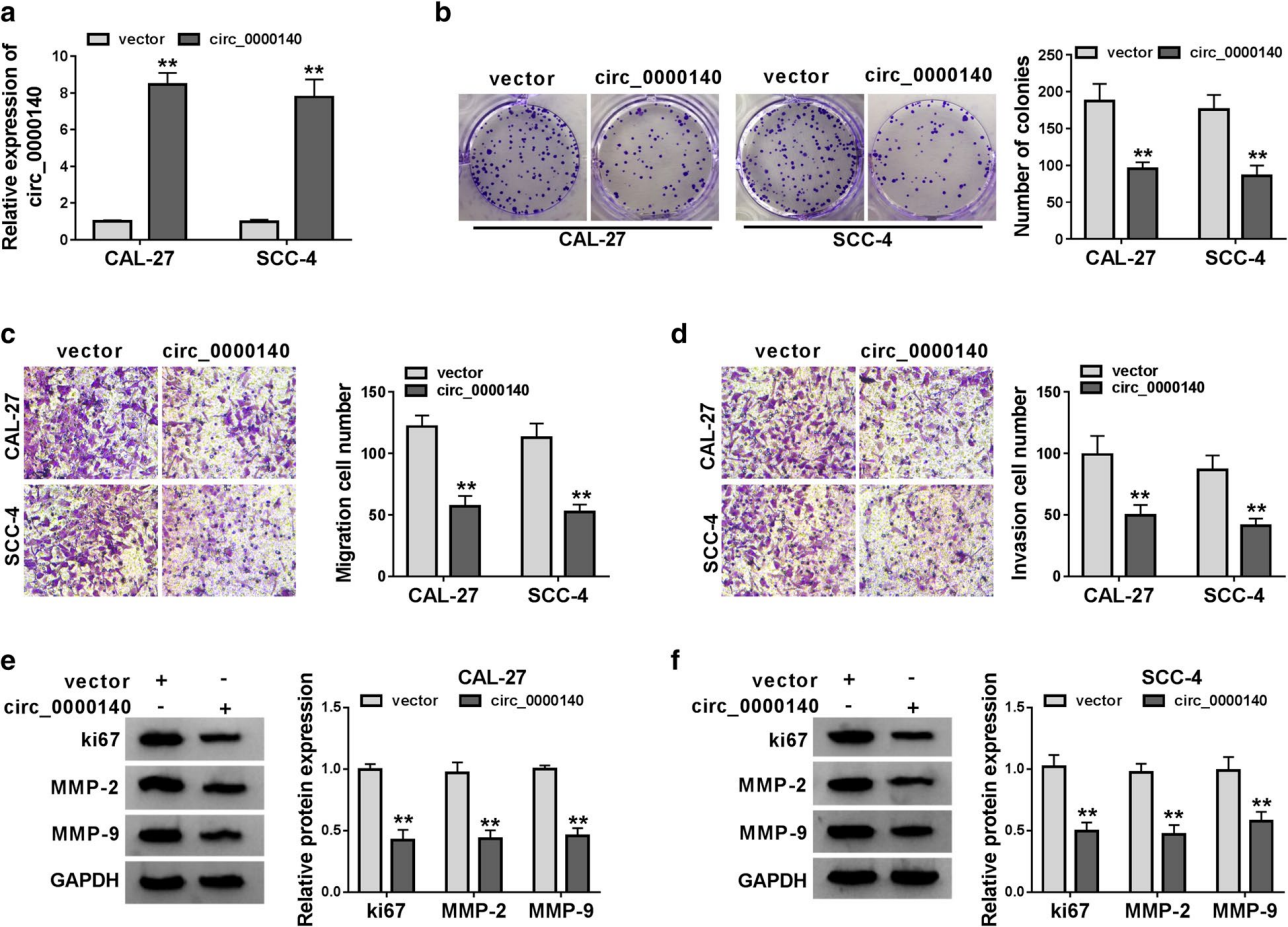
Effects of circ_0000140 overexpression on the proliferation, migration and invasion of OSCC cells. CAL-27 and SCC-4 cells were transfected with circ_0000140 overexpression vector or vector. **a** The expression of circ_0000140 in CAL-27 and SCC-4 cells was detected by qRT-PCR to evaluate transfection efficiency. **b** Colony formation assay was performed to measure the number of colonies in CAL-27 and SCC-4 cells. **c**, **d** The number of migrated and invaded CAL-27 and SCC-4 cells was determined by transwell assay. **e**, **f** WB analysis was performed to detect the protein levels of ki67, MMP-2 and MMP-9 in CAL-27 and SCC-4 cells. ** *P* < 0.01

To explore the role of circ_0000140 on OSCC progression, we used circ_0000140 overexpression vector to conduct gain-functional experiments. The increase of circ_0000140 expression in CAL-27 and SCC-4 cells revealed that the transfection of circ_0000140 overexpression vector was successful (Fig. 2a). Subsequently, we measured the proliferation, migration and invasion of OSCC cells. Colony formation assay results indicated that circ_0000140 overexpression reduced the number of colonies in CAL-27 and SCC-4 cells, suggesting that its overexpression inhibited the proliferation of OSCC cells (Fig. 2b). Meanwhile, transwell assay results showed that overexpressed circ_0000140 markedly suppressed the number of migrated and invaded CAL-27 and SCC-4 cells, indicating that circ_0000140 overexpression restrained the migration and invasion abilities of OSCC cells (Fig. 2c, d). The detection results of proliferation marker protein ki67 and metastasis marker proteins MMP-2 and MMP-9 showed that the protein levels of ki67, MMP-2 and MMP-9 were significantly inhibited by circ_0000140 overexpression in CAL-27 and SCC-4 cells, which once again confirmed that circ_0000140 overexpression could hinder the proliferation and metastasis of OSCC cells (Fig. 2e, f). All data revealed that circ_0000140 expression had a negative effect on OSCC development.

### Overexpressed circ_0000140 inhibited the glycolysis metabolism of OSCC cells

**Fig. 3:**
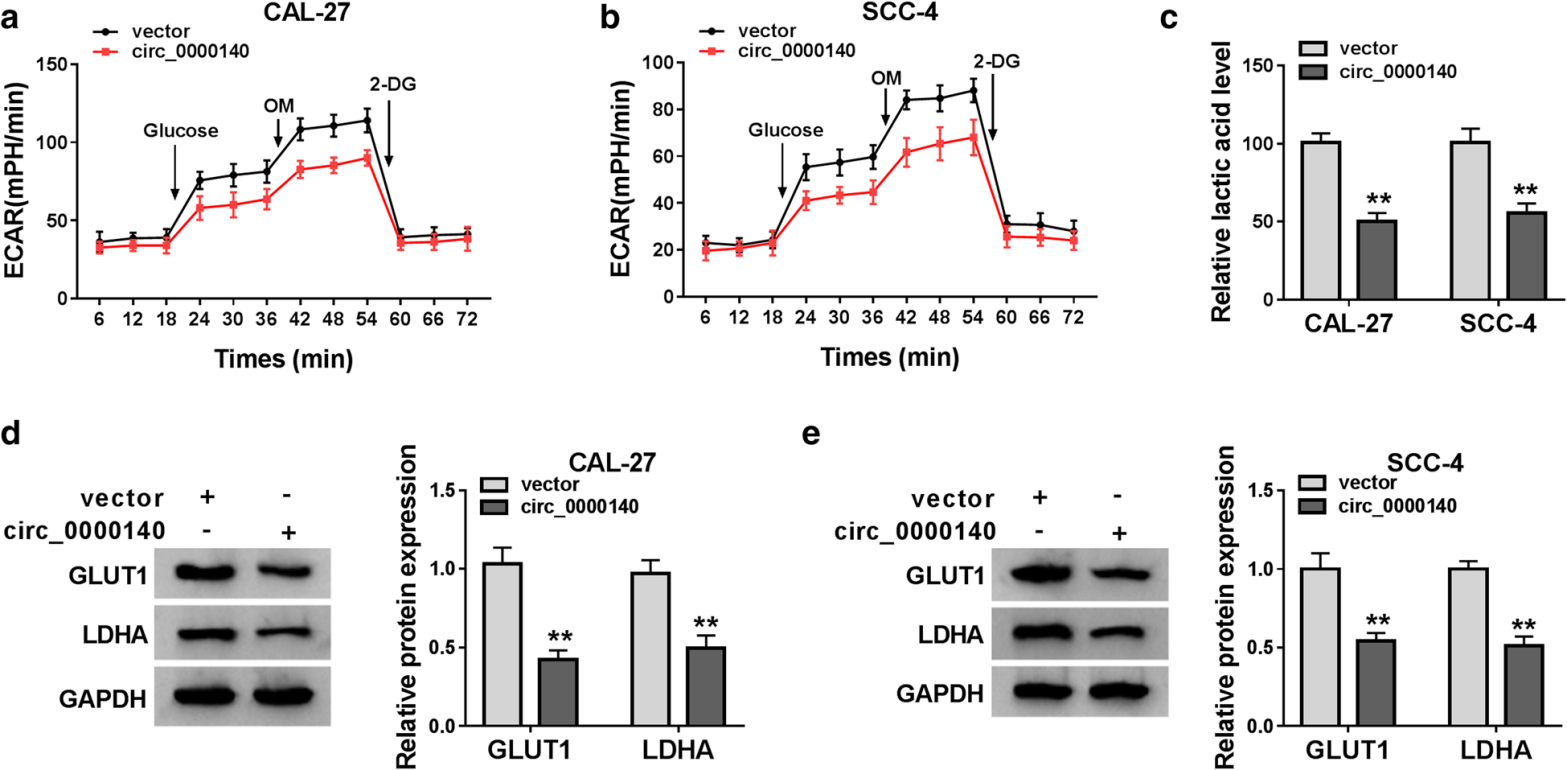
Effects of circ_0000140 overexpression on the glycolysis metabolism of OSCC cells. CAL-27 and SCC-4 cells were transfected with circ_0000140 overexpression vector or vector. **a**, **b** The ECAR of CAL-27 and SCC-4 cells was measured by Seahorse XF Extracellular Flux Analyzer. **c** The lactate acid level of CAL-27 and SCC-4 cells was tested by Lactate Assay Kit. **d**, **e** The protein levels of GLUT1 and LDHA in CAL-27 and SCC-4 cells were detected by WB analysis. ** *P* < 0.01

To test the effect of circ_0000140 on glycolysis metabolism in OSCC cells, we measured the ECAR of CAL-27 and SCC-4 cells. As shown in Fig. 3a, b, the ECAR of CAL-27 and SCC-4 cells was markedly reduced in the circ_0000140 overexpression group compared with that in the control group. Through detecting the lactate acid level of CAL-27 and SCC-4 cells, we discovered that overexpression of circ_0000140 remarkably hindered the lactate acid level of OSCC cells (Fig. 3c). Furthermore, we also examined the protein levels of glycolysis related genes GLUT1 and LDHA, and found that circ_0000140 overexpression significantly restrained the protein levels of GLUT1 and LDHA in CAL-27 and SCC-4 cells (Fig. 3d, e). These data indicated that circ_0000140 could also affect the glycolysis metabolism of OSCC.

### Circ_0000140 could absorb miR-182-5p in OSCC cells

**Fig. 4:**
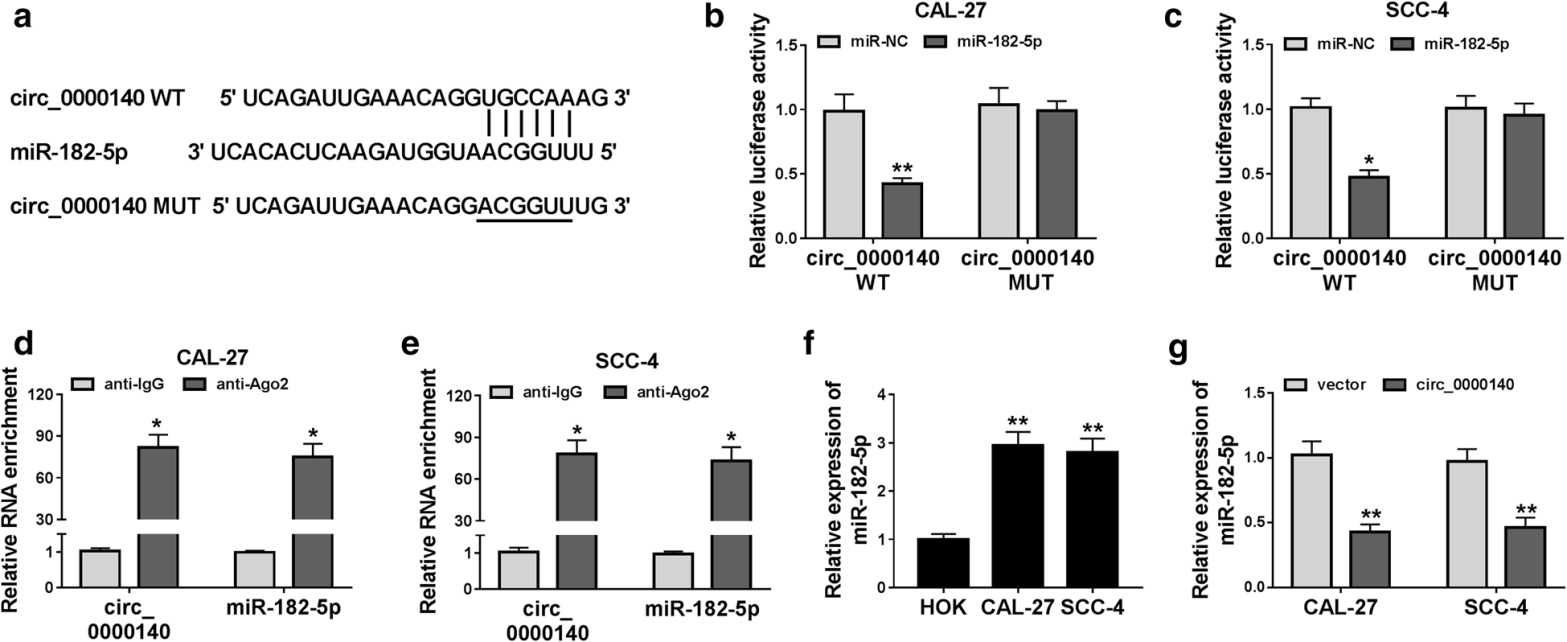
Circ_0000140 could absorb miR-182-5p. **a** The sequences of circ_0000140 containing the miR-182-5p binding sites or mutant binding sites were presented. **b**, **c** Dual-luciferase reporter assay was used to detect the interaction between circ_0000140 and miR-182-5p in CAL-27 and SCC-4 cell. **d**, **e** The enrichment of circ_0000140 and miR-182-5p in anti-Ago2 or anti-IgG was measured by the RIP assay. **f** qRT-PCR was performed to measure the expression of miR-182-5p in OSCC cells (CAL-27 and SCC-4) and NOK cells. **g** The expression of miR-182-5p in CAL-27 and SCC-43 cells was assessed by qRT-PCR to assess the effect of circ_0000140 overexpression on miR-182-5p expression. * *P* < 0.05, ** *P* < 0.01

For perfecting the molecular mechanism of circ_0000140 function as a “miRNA sponge”, we used the Circinteractome tool to predict the miRNA and found that miR-182-5p could bind complementarily to circ_0000140 (Fig. 4a). To further validate the binding ability between them, we built the circ_0000140 WT and circ_0000140 MUT reporter vectors to perform dual-luciferase reporter assay. The results suggested that miR-182-5p overexpression markedly suppressed the luciferase activity of circ_0000140 WT reporter vector in CAL-27 and SCC-4 cells, but not effect on the luciferase activity of circ_0000140 MUT reporter vector (Fig. 4b, c). Besides, RIP assay results also revealed that circ_0000140 and miR-182-5p were significantly enriched in anti-Ago2 compared with that in anti-IgG, suggesting that circ_0000140 might be related to miR-182-5p (Fig. 4d, e). Moreover, we also detected the expression of miR-182-5p in CAL-27 and SCC-4 cells and discovered the fact that miR-182-5p was markedly upregulated in OSCC cells compared with that in HOK cells (Fig. 4f). To further explore the effect of circ_0000140 on miR-182-5p expression, we detected the miR-182-5p expression in CAL-27 and SCC-4 cells transfected with circ_0000140 overexpression vector. The results indicated that miR-182-5p expression was inhibited by circ_0000140 overexpression in OSCC cells (Fig. 4g). Hence, our results proved that miR-182-5p could be sponged by circ_0000140 in OSCC.

### MiR-182-5p mimic could revert the suppression effect of circ_0000140 overexpression on the progression of OSCC cells

**Fig. 5:**
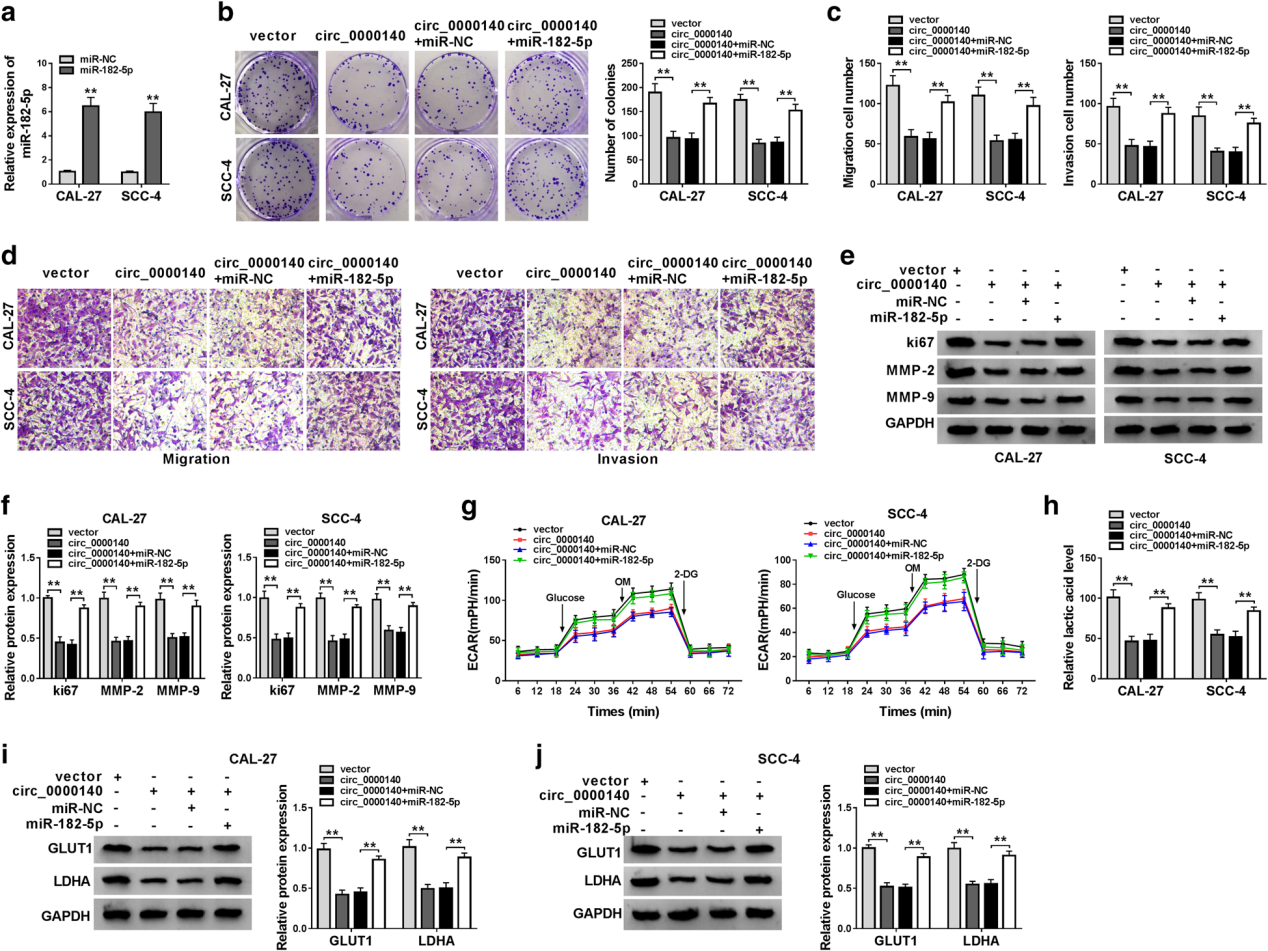
Effects of miR-182-5p mimic on the progression of OSCC cells. **a** The expression of miR-182-5p in CAL-27 and SCC-4 cells was measured by qRT-PCR to evaluate the transfection efficiency of miR-182-5p mimic. CAL-27 and SCC-4 cells were co-transfected with circ_0000140 overexpression vector and miR-182-5p mimic. **b** The number of colonies in CAL-27 and SCC-4 cells was detected by colony formation assay. **c**, **d** The number of migrated and invaded CAL-27 and SCC-4 cells was tested by transwell assay. **e**, **f** The protein levels of ki67, MMP-2 and MMP-9 in CAL-27 and SCC-4 cells were assessed by WB analysis. **g** Seahorse XF Extracellular Flux Analyzer was used to evaluate the ECAR of CAL-27 and SCC-4 cells. **h** The lactate acid level of CAL-27 and SCC-4 cells was determined by Lactate Assay Kit. **i**, **j** WB analysis was performed to measure the protein levels of GLUT1 and LDHA in CAL-27 and SCC-4 cells. ** *P* < 0.01

To confirm the function of miR-182-5p, we conducted rescue experiments using miR-182-5p mimic. First, we tested the transfection efficiency of miR-182-5p mimic, and the enhanced effect of miR-182-5p mimic on miR-182-5p expression indicated that its transfection efficiency was good (Fig. 5a). Then, we co-transfected circ_0000140 overexpression vector and miR-182-5p mimic into CAL-27 and SCC-4 cells. Through colony formation and transwell assays, we found that miR-182-5p overexpression could recover the inhibition effect of circ_0000140 overexpression on the number of colonies, migrated and invaded CAL-27 and SCC-4 cells (Fig. 5b–d). Also, the suppression effect of circ_0000140 overexpression on the protein levels of ki67, MMP-2 and MMP-9 in CAL-27 and SCC-4 cells also could be inverted by miR-182-5p overexpression (Fig. 5e, f). Moreover, we measured the ECAR, lactate acid level and the protein levels of GLUT1 and LDHA to assess the function of miR-182-5p on the glycolysis metabolism of OSCC cells. The results showed that miR-182-5p overexpression also reversed the inhibitory effect of overexpressed circ_0000140 on the ECAR, lactate acid level and the protein levels of GLUT1 and LDHA in CAL-27 and SCC-4 cells, suggesting that miR-182-5p overexpression inverted the suppression of circ_0000140 overexpression on the glycolysis metabolism of OSCC cells (Fig. 5 g–J). These results further confirmed the importance of miR-182-5p in the regulation of circ_0000140 on OSCC development.

### MiR-182-5p could target CDC73 in OSCC cells

**Fig. 6:**
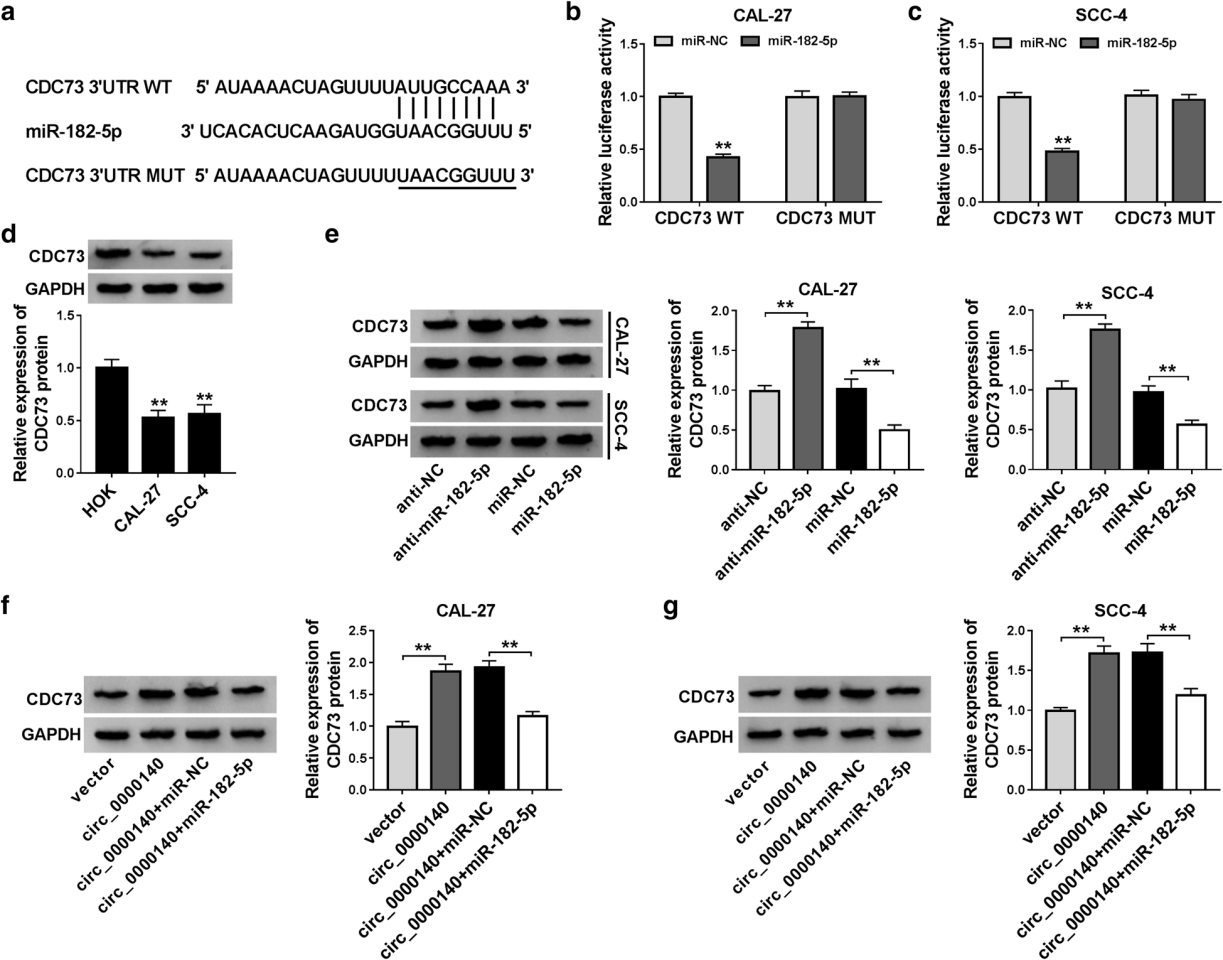
MiR-182-5p could target CDC73. **a** The sequences of CDC73 3′UTR containing the miR-182-5p binding sites or mutant binding sites were shown. **b**, **c** The interaction between miR-182-5p and CDC73 in CAL-27 and SCC-4 cells was assessed by dual-luciferase reporter assay. **d** The protein level of CDC73 in OSCC cells (CAL-27 and SCC-4) and HOK cells was determined by WB analysis. **e** WB analysis was used to measure the CDC73 protein level in CAL-27 and SCC-4 cells to evaluate the miR-182-5p expression on CRC73 expression. **f**, **g** The CDC73 protein level in CAL-27 and SCC-4 cells was detected by WB analysis to evaluate the circ_0000140 and miR-182-5p expression on CDC73 expression. ** *P* < 0.01

Meanwhile, we used the TargetScan tool to predict the downstream target of miR-182-5p and discovered that CDC73 3′UTR had a complementary binding site with miR-182-5p (Fig. 6a). Dual-luciferase reporter assay results determined that miR-182-5p mimic remarkably hindered the luciferase activity of CDC73 WT reporter vector in CAL-27 and SCC-4 cells, while did not affect CDC73 MUT reporter vector (Fig. 6b, c). Through detecting the CDC73 expression in OSCC cells, we found that the CDC73 protein level was markedly decreased in CAL-27 and SCC-4 cells (Fig. 6d). Besides, we also investigated the effect of miR-182-5p expression on CDC73 expression. As shown in Fig. 6e, the CDC73 protein level was promoted by miR-182-5p inhibition, while suppressed by miR-182-5p overexpression. At the same time, we measured the CDC73 expression in CAL-27 and SCC-4 cells co-transfected with circ_0000140 overexpression vector and miR-182-5p mimic. The results indicated that circ_0000140 overexpression could increase the CDC73 protein level, while this promotion could be reversed by miR-182-5p mimic, suggesting that CDC73 expression was regulated by circ_0000140 and miR-182-5p (Fig. 6f, g). Therefore, these results suggested that CDC73 is a target of miR-182-5p.

### Knockdown of CDC73 reversed the suppression effect of miR-182-5p inhibitor on the progression of OSCC cells

**Fig. 7:**
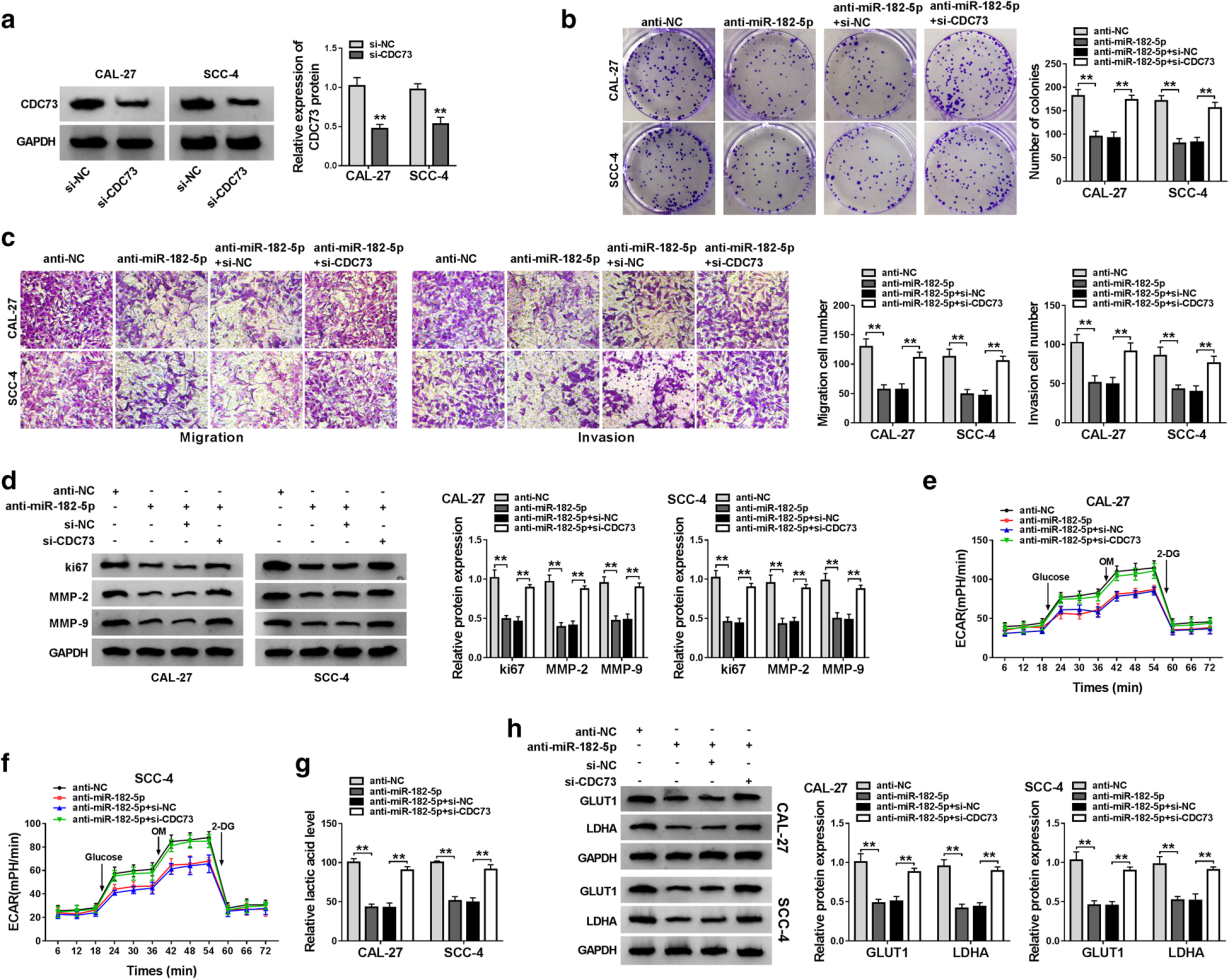
Effects of CDC73 silencing on the progression of OSCC cells. **a** The protein level of CDC73 in CAL-27 and SCC-4 cells was measured by WB analysis to evaluate the transfection efficiency of si-CDC73. CAL-27 and SCC-4 cells were co-transfected with anti-miR-182-5p and si-CDC73. **b** Colony formation assay was performed to assess the number of colonies in CAL-27 and SCC-4 cells. **c** The number of migrated and invaded CAL-27 and SCC-4 cells was detected by transwell assay. **d** WB analysis was used to measure the protein levels of ki67, MMP-2 and MMP-9 in CAL-27 and SCC-4 cells. **e**, **f** Seahorse XF Extracellular Flux Analyzer was employed to assess the ECAR of CAL-27 and SCC-4 cells. **g** The lactate acid level of CAL-27 and SCC-4 cells was detected by Lactate Assay Kit. **h** The protein levels of GLUT1 and LDHA in CAL-27 and SCC-4 cells were tested by WB analysis. ** *P* < 0.01

To further verify that CDC73 was the target gene of miR-182-5p, we performed the rescue experiments using si-CDC73. Significant inhibition of the CDC73 protein level by si-CDC73 indicated that its transfection efficiency was good (Fig. 7a). Then, we co-transfected anti-miR-182-5p and si-CDC73 into CAL-27 and SCC-4 cells. Colony formation and transwell assays revealed that anti-miR-182-5p inhibited the proliferation, migration and invasion of CAL-27 and SCC-4 cells, while this inhibition effect could be reversed by CDC73 silencing (Fig. 7b, c). The recovery effect of CDC73 knockdown on the protein levels of ki67, MMP-2 and MMP-9 also confirmed that silenced CDC73 could invert the inhibitory effect of miR-182-5p inhibitor on the proliferation, migration and invasion of OSCC cells (Fig. 7d). Through detection of the ECAR, lactic acid level and the GLUT1 and LDHA protein levels, we uncovered that miR-182-5p inhibitor could restrain the ECAR, lactic acid level and reduce the protein levels of GLUT1 and LDHA to suppress the glycolysis metabolism of OSCC cells. However, the silencing of CDC73 also could recover the suppression of the miR-182-5p inhibitor on the glycolysis metabolism of OSCC cells (Fig. 7e–h). Therefore, these results confirmed that CDC73 had a vital function on the regulation of the circ_0000140/miR-182-5p axis on OSCC progression.

### Circ_0000140 overexpression reduced the tumor growth of OSCC in vivo

**Fig. 8:**
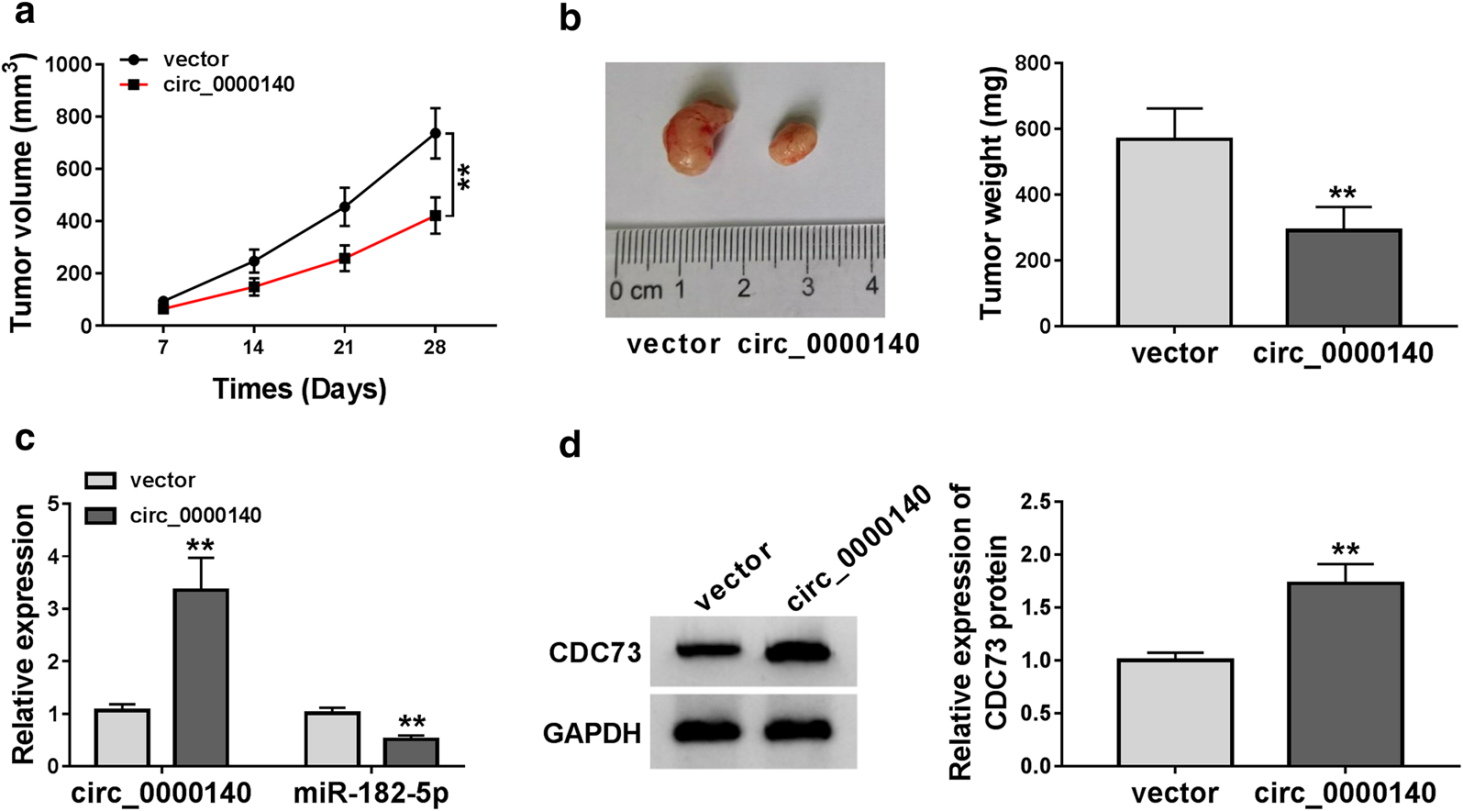
Effects of circ_0000140 overexpression on the tumor growth of OSCC in vivo. **a** Tumor volume was calculated at the indicated time points (7 d, 14 d, 21 d, and 28 d). **b** Tumor weight was measured after removed the tumors from mice. **c** The expression levels of circ_0000140 and miR-182-5p were tested by qRT-PCR. **d** WB analysis was used to assess the protein level of CDC73 in the tumors. ** *P* < 0.01

For investigating the function of circ_0000140 in OSCC in vivo, we constructed the OSCC mice xenograft models. After 28 days of measurement, we found that the growth rate of tumor volume was significantly inhibited in the circ_0000140 overexpression group (Fig. 8a). Through the measurement of tumor weight, we uncovered that the tumor weight of the circ_0000140 overexpression group was markedly reduced (Fig. 8b). To confirm that the transfection of circ_0000140 overexpression vector was successful, we detected the circ_0000140 expression and discovered that circ_0000140 expression was indeed promoted in the circ_0000140 overexpression group (Fig. 8c). Meanwhile, we also measured the expression of miR-182-5p and CDC73 expression in tumor tissues and found that in the circ_0000140 overexpression group, miR-182-5p expression was remarkably inhibited and the CDC73 protein level was significantly increased (Fig. 8c, d). Hence, these data indicated that circ_0000140 might play an anti-cancer role by regulating the miR-182-5p/CDC73 axis.

## Discussion

At present, the treatment of OSCC is still based on the principle of early detection, early diagnosis, and early treatment. However, because the symptoms of early OSCC are generally not obvious, the diagnosis is usually advanced, which greatly increases the mortality rate of OSCC patients [25]. CircRNAs are considered an ideal marker for many cancers, mainly because its expression is closely related to cancer progression [26, 27]. However, the research of circRNAs on the progress of OSCC is still in the exploratory stage, and there are still a lot of new circRNAs waiting for us to explore. Here, we explored a newly discovered circRNA, circ_0000140, which had attracted our attention due to it had downregulated expression in OSCC patients [13, 14]. We also discovered that circ_0000140 had a decreased expression in OSCC, and it was a stable cyclic transcript and mainly located in the cytoplasm.

Generally speaking, cancer occurs mainly because cell mutation causes cell abnormal proliferation, usually accompanied by metastasis and spread [28]. The glycolysis metabolism of cells is the key to cellular energy production, which is essential for the normal operation of cells [29]. Therefore, exploring the proliferation, metastasis and glycolysis metabolism capacities of cancer cells can well explain the progress of cancer. In our research, we found that circ_0000140 overexpression could suppress the proliferation, migration, invasion and glycolysis metabolism of OSCC, which proved its anti-cancer effect in OSCC. In vivo experiments, we also proved that it could inhibit the tumor growth of OSCC. All data was similar with the results of Peng et al. [14]. Further experiments showed that circ_0000140 could absorb miR-182-5p to regulate CDC73 expression. This evidence confirmed that circ_0000140 might act as a tumor suppressor to participate in the regulation of OSCC progression.

It is reported that knockdown of LINC00173 elevated the miR-182-5p expression to promote the proliferation, migration and suppressed the apoptosis of lung cancer [30]. And circRNA BCRC-3 hindered the proliferation of bladder cancer through sponging miR-182-5p [31]. These confirmed that the high expression of miR-182-5p played a vital role in cancer development. Herein, we found that miR-182-5p was upregulated in OSCC, which was consistent with the results of Guo et al. and Li et al. [21, 22]. The reverse effect of miR-182-5p overexpression on the inhibition of circ_0000140 on OSCC progression confirmed that miR-182-5p could be absorbed by circ_0000140. Although tumor suppressor CDC73 has been less studied in OSCC, its low expression in other cancers and its anti-cancer effect have been widely confirmed [23, 32]. We concluded that CDC73 expression was regulated by circ_0000140 and miR-182-5p in OSCC, and its silencing could invert the suppression of miR-182-5p inhibitor on OSCC progression.

Our results provided a key target for the treatment of OSCC. The inhibitory effect of circ_0000140 overexpression and miR-182-5p inhibition on OSCC progression could provide a theoretical basis for the treatment of OSCC. In addition, the discovery of new circ_0000140 has enriched the studies related to circRNA, but the role and mechanism of circ_0000140 in other cancers is still worth further discussion.

## Conclusions

In summary, we suggested that circ_0000140 was under-expressed in OSCC, and it could hinder the proliferation, migration, invasion and glycolysis metabolism of OSCC through regulating the miR-182-5p/CDC73 axis. These findings might help us better understand the molecular mechanisms of OSCC progression to perfect the circRNA-targeted therapies of OSCC.

## Data Availability

The datasets used and/or analyzed during the current study are available from the corresponding author on reasonable request.

## Abbreviations

OSCC: Oral squamous cell carcinoma
CDC73: Cell division cycle 73
HOK: Human normal oral epithelial keratinocytes
ATCC: American Type Culture Collection
FBS: Fetal bovine serum
WB: Western blot
ECAR: Extracellular acidification rate
OM: Oligomycin
RIP: RNA immunoprecipitation
